# Global spatial ecology of three closely-related gadfly petrels

**DOI:** 10.1038/srep23447

**Published:** 2016-03-22

**Authors:** Raül Ramos, Iván Ramírez, Vitor H. Paiva, Teresa Militão, Manuel Biscoito, Dília Menezes, Richard A. Phillips, Francis Zino, Jacob González-Solís

**Affiliations:** 1 Institut de Recerca de la Biodiversitat (IRBio) and Departament de Biologia Animal, Universitat de Barcelona, Av Diagonal 643, Barcelona 08028, Spain; 2 BirdLife International–The David Attenborough Building, Pembroke St, Cambridge, CB2 3QZ, United Kingdom; 3 Marine and Environmental Sciences Centre (MARE), Department of Life Sciences, University of Coimbra, 3004-517 Coimbra, Portugal; 4 Marine and Environmental Sciences Centre (MARE), Estação de Biologia Marinha do Funchal and OOM-Museu de História Natural do Funchal, Rua da Mouraria 31, Funchal 9004-546, Madeira, Portugal; 5 Parque Natural da Madeira, Quinta do Bom Sucesso, Caminho do Meio, Funchal 9050-251, Madeira, Portugal; 6 British Antarctic Survey (BAS), Natural Environment Research Council, High Cross, Madingley Road, Cambridge CB3 0ET, United Kingdom; 7 Freira Conservation Project (FCP), Avenida do Infante 26, 9000-015 Funchal, Madeira, Portugal

## Abstract

The conservation status and taxonomy of the three gadfly petrels that breed in Macaronesia is still discussed partly due to the scarce information on their spatial ecology. Using geolocator and capture-mark-recapture data, we examined phenology, natal philopatry and breeding-site fidelity, year-round distribution, habitat usage and at-sea activity of the three closely-related gadfly petrels that breed in Macaronesia: Zino’s petrel *Pterodroma madeira*, Desertas petrel *P. deserta* and Cape Verde petrel *P. feae*. All *P. feae* remained around the breeding area during their non-breeding season, whereas *P. madeira* and *P. deserta* dispersed far from their colony, migrating either to the Cape Verde region, further south to equatorial waters in the central Atlantic, or to the Brazil Current. The three taxa displayed a clear allochrony in timing of breeding. Habitat modelling and at-sea activity patterns highlighted similar environmental preferences and foraging behaviours of the three taxa. Finally, no chick or adult was recaptured away from its natal site and survival estimates were relatively high at all study sites, indicating strong philopatry and breeding-site fidelity for the three taxa. The combination of high philopatry, marked breeding asynchrony and substantial spatio-temporal segregation of their year-round distribution suggest very limited gene flow among the three taxa.

From an ecological perspective, assessing year-round phenology, distribution, habitat use and behaviour in closely related taxa is important for understanding reproductive isolation mechanisms, and therefore potential for lineage divergence. Currently much research to identify evolutionary significant units and define appropriate taxonomic boundaries among populations focusses on population genetic structure and gene flow^1^,^2^. However, recently diverged species may not have acquired large genetic differences or became phenotypically distinguishable, diagnosable and reproductively incompatible, and as a consequence their taxonomic relationships are often difficult to tease apart^3^. In this regard, assessing ecological divergence of closely-related species in multiple traits is crucial for a thorough understanding of the mechanisms underlying the genetic structuring of populations^4^, and can also help identify evolutionary significant units and defining taxonomic boundaries, which is key to assessing their conservation status^5^,^6^,^7^.

In pelagic ecosystems, seabirds provide some interesting challenges when aiming to disentangle factors influencing population structure, since they have one characteristic that promotes, and another that opposes genetic divergence: a high degree of philopatry *vs.* a huge potential for dispersal because of their capacity for long-distance flight. For instance, in many closely-related seabird species, spatially-separated populations show slight but obvious geographic differences in plumage, morphology and vocalizations^8^,^9^. In the absence of obvious physical barriers in the open ocean, habitat specialization and local adaptations to specific oceanographic conditions may nevertheless promote ecological differentiation and, ultimately, speciation. Recent phylogeographic studies on marine predators have suggested three non-exclusive intrinsic factors that may cause population differentiation^4^,^10^: high philopatry, breeding asynchrony and year-round spatio-temporal segregation in foraging areas.

Despite considerable research effort, several recent studies of demography and phenology have failed to conclusively determine the taxonomic relationships within a number of seabird species complexes. One solution is to take advantage of recent advances in biologging, such as the use of geolocators (Global Location Sensors [GLS] loggers), for tracking of multiple individuals from different populations, which provides new insights into year-round spatial ecology and ecological divergence of closely-related taxa. Migration schedules (dates of arrival and departure from breeding and non-breeding grounds), distribution and foraging activity at sea, can now be assessed with such loggers. In addition, understanding the timing and patterns of spatial movements of predators is not only fundamental to exploring their evolutionary ecology, but also key information for ensuring their conservation. For instance, determining the degree of spatio-temporal overlap among species or populations is essential for assessing their exposure to common threats^11^.

The gadfly petrels (genus *Pterodroma*) are some of the most threatened and least known of all seabirds, as they often breed in remote islands that are difficult to access and in small numbers, returning to colonies only at night^10^,^12^. In addition, the presence of cryptic taxa has further conservation implications^13^, as taxonomic splitting leads to smaller effective population sizes which may face localised threats^12^. The gadfly petrels of Macaronesia are a case in point; they occur in the tropical and subtropical pelagic ecosystems of the Atlantic Ocean, and despite recent studies, the degree of genetic and ecological divergence is still debated. Taxa within this complex were once considered subspecies of the widely-distributed soft-plumaged petrel, *P. mollis*^14^. Even in the absence of obvious morphological and vocal differentiation among the different breeding populations in Macaronesia^15^,^16^, after recent, long debate, the general consensus seems to be that the three taxa are independently-evolving lineages that may warrant specific status^17^: Zino’s petrel (*P. madeira*) breeding on Madeira Island, Desertas petrel (*P. deserta*) on Bugio Island (in Desertas Islands, only 50 km apart from Madeira Island and belonging to the Madeira archipelago) and Cape Verde petrel (*P. feae*) on four islands of the Cape Verde archipelago. Several phylogenetic studies concluded that the Madeiran archipelago has been colonised twice by ancestral petrels from Cape Verde: a first establishment in Madeira Island during the early Pleistocene and a second colonisation of the Desertas Islands during the late Pleistocene^18^,^19^. This scenario (hypothesised previously by Bourne^20^) would explain the current breeding distribution as well as the slight morphological differences between the populations despite the close proximity of some of the breeding sites. However, more recent molecular evidence (based on mitochondrial and nuclear genes) suggested a different phylogenetic scenario, with more recent divergence times for the three taxa and with *P. madeira* at the basis of the gene tree^17^. Thus, given the phylogenetic and taxonomic uncertainties, complementary information on non-breeding distribution and behaviour at-sea is key for assessing potential adaptations to specific habitats or conditions, and the role of ecological segregation in the differentiation of such taxa. This information has major implications for conservation as taxonomic delimitation between differentiated populations would reduce their effective population sizes, and thus increase their threat status, requiring greater overall conservation efforts^13^.

In this study, using miniaturized geolocators, we compare year-round distribution, marine habitat use and foraging activity, and hence provide new information for evaluating the ecological divergence of the three Macaronesian gadfly petrels. For each taxon, we specifically aimed to (1) define accurate breeding and migration schedules, (2) identify their main breeding and non-breeding foraging grounds and assess spatio-temporal overlap, (3) characterize the marine habitat in such areas, and finally, (4) provide new insights into their activity patterns. In addition, we present Capture-Mark-Recapture (CMR) data on the three species that allowed us to investigate the degree of natal philopatry and breeding-site fidelity.

## Methods

### Study species and sampling design

The three closely-related gadfly petrels inhabit Madeira and Cape Verde archipelagos and surrounding pelagic waters (Table 1)^21^. All three are small, grey and white petrels, with wingspans of 800–843 mm in *P. madeira*, 860–940 mm in *P. deserta*, and 880–943 mm in *P. feae*^15^. They are long-lived, colonial, breed annually in rock crevices and burrows excavated on remote cliffs and steep slopes, and visit their nests mainly on dark nights. Breeding seasons are long (*ca.* six months), and known to differ between the species; *P. madeira* and *P. deserta* breed during the northern summer and early winter, while *P. feae* breeds throughout the winter months (see Table 1 for approximate schedules). Little is known about their diet and foraging tactics, although it is assumed that they mainly feed on small squid and pelagic fish^21^. The three taxa are of conservation concern and all have small population sizes (Table 1)^22^.

The present study was conducted at three breeding colonies in the archipelagos of Madeira (Madeira Island and Bugio Island) and Cape Verde (Fogo Island), over eight years (2007–2014). At each colony, breeding adults incubating an egg or rearing a chick were fitted with a small, leg-mounted, combined geolocator-immersion logger (Mk14 [British Antarctic Survey, BAS] for *P. madeira* and *P. deserta* and Biotrack Mk3005 [formerly BAS Mk 19] for *P. feae*, weighing 1.5 and 2.5 g, respectively, and corresponding to 0.5–0.9% of bird body mass; thus well below the threshold of 3% above which deleterious effects are more likely to occur^23^. These loggers recorded elapsed time, light intensity and saltwater immersion.

In addition to the logger deployments, specific Capture-Mark-Recapture (CMR) programmes were carried out at each study site (Table 2): breeding *P. madeira* and *P. deserta* were captured in burrows, while *P. feae* were captured using vertical mist nets (15 m nets with 4 pockets and 20 × 20 mm mesh). Nets around the Cape Verde colony were opened after sunset and checked frequently in order to minimize the time that birds remained trapped. Every individual captured for the first time was measured and ringed with a monel band; recaptures of ringed individuals were also recorded. We modelled capture and survival probabilities of the three populations of gadfly petrels separately (specific date surveys, CMR methods and model selection are described and shown in the electronic supplementary material: Table S1 and Table S2). Survival probability was used to infer breeding-site fidelity and evaluate emigration rate from the capture site. Additionally, chicks captured in burrows were ringed each year around the time of fledgling to infer natal philopatry (although only for a few burrows in *P. feae*; Table 2).

All animals were handled in strict accordance with good animal practice as defined by the current European legislation, and all animal work was approved by the respective regional committees for scientific capture (Direcção Geral do Ambiente, Government of Cape Verde: 01/2009, 02/2010, 01/2011, 01/2012, 04/2013 and 018/2014, and the Madeira Natural Park Service: 394/2008/CAPT, 432/2009/CAPT, 544/2010/CAPT, and 584/2011/CAPT).

### Spatial and activity data and their analyses

Two positions per day (local midday and midnight) were estimated from the light data using the BASTrak software suite^24^, with an average error of ~200 km (or ~ 2°)^25^. From filtered data, we estimated five phenological and spatial parameters for every complete migration cycle (see Table S3 in the electronic supplementary material): (1) departure date, (2) arrival date, (3) duration of the non-breeding period (in days), (4) non-breeding range (orthometric distance between the breeding colony and the average of all locations within the 5% Utilization Distribution [UD]; in km), and (5) latitude of the centroid of the core of the non-breeding distribution (mean latitude of all positions within the 5% UD; in degrees). We evaluated the effect of species on these non-breeding parameters using generalized linear mixed models (GLMMs): the above five parameters were the response variables, species was the fixed effect, and year of sampling was the random effect in every model (Table 3). Filtering methods for the spatial data, the main migratory characteristics and GLMM selection procedures are fully described in the electronic supplementary material.

The 25%, 50%, 75% and 95% UDs (25–95% UD; in km^2^; *kernelUD* function in the adehabitat v.1.8.7 package in R)^26^ were calculated using the filtered, smoothed locations for each species, separately for the breeding and non-breeding periods. Finally, we calculated the spatial overlap between the areas (95% UDs) used during the breeding and non-breeding periods both within and between species (*i.e.* species^*^period; Table 4) using the *kerneloverlap* function in the *adehabitat* package (VI method)^27^.

The loggers also tested for immersion in sea water every 3 s using 2 electrodes, and provided a value (0 to 200) corresponding to the sum of positive tests in each 10-min period, which can be transformed to the proportion of time spent wet, *i.e.*, when the bird was sitting on the sea surface or diving. As activity budgets vary seasonally in many seabird species, we first modelled the dynamics of time spent flying throughout the annual cycle in the three species using GLMMs (Table S4). Secondly, we evaluated the effect of moonlight levels on the flight activity of each species throughout the year (Table S5). For plotting purposes, activity budgets were also modelled using generalized additive mixed models (GAMMs). Finally, following Dias *et al*.^28^, we calculated a ‘night flight index’ (NFI) as the difference between the proportion of time spent flying during darkness and daylight, divided by the higher of these two values. This index ranges from -1 (flight activity restricted to daylight) to 1 (flight restricted to night). Filtering methods for the activity data, and GLMM and GAMM selection procedures are described in the electronic supplementary material.

Processing of the environmental data and habitat modelling are detailed in the electronic supplementary material. Briefly, habitat suitability models were developed for the tracked birds using the MaxEnt v.3.3.3e software^29^, a program for modelling ecological niches from presence-only species records. Habitat models were run with six non-redundant variables (seafloor depth [BAT, m], the gradient for BAT, surface chlorophyll *a* concentration [CHLa, mg m^−3^], sea surface temperature [SST, °C], the gradient for SST, and wind speed [WIND, m s^−1^]) for each of the ten data subsets, including specific breeding, non-breeding and year-round subsets, plus a supra-specific global subset. The results were summarized as the average of the 100 models, and model evaluation was performed using the area under the curve (AUC) statistic, which measures the ability of model predictions to discriminate seabird presence from background points (Table 5). In the results, all means are presented ± standard deviation, unless otherwise stated.

## Results

### Capture-Mark-Recapture (CMR) data

A total of 64 *P. madeira*, 217 *P. deserta* and 128 *P. feae* were ringed as adults and monitored annually thereafter (Table 2) at each breeding colony. A total of 155 chicks of *P. madeira*, 170 of *P. deserta* and 6 of *P. feae* were ringed at the time of fledgling, and 6, 8 and 1 of them, respectively, were recaptured in the same colony some years later. Neither chicks nor adults were ever recaptured in a colony different from that in which they were ringed (Table 2).

Model selection for each species is fully described in the electronic supplementary material and CMR results are summarised in Table 2. Similarly for the three species, the goodness-of-fit test revealed neither a significant difference in survival between newly-marked and previously-marked birds (Test 3SR) nor significant trap-dependence (Table S1 in the electronic supplementary material). The models with the greatest weight in the three species had time-specific recapture probability and constant survival (Table S2 in the electronic supplementary material). On average, adult recapture probabilities were estimated as 0.468 ± 0.143 for *P. madeira* (mean ± SD), 0.393 ± 0.217 for *P. deserta* and 0.296 ± 0.163 for *P. feae* (Table 2). Adult survival estimates for each of the species were made under model Φ (*·*) p (*t)*: 0.830 ± 0.052 for *P. madeira* (95% confidence interval [CI]: 0.703–0.910), 0.745 ± 0.031 for *P. deserta* (95% CI: 0.681–0.800) and 0.797 ± 0.042 for *P. feae* (95% CI: 0.702–0.868).

### Phenology, migration characteristics and year-round distribution

We obtained 37 complete tracks from 31 individual gadfly petrels for their breeding and non-breeding seasons (8 for *P. madeira*, 16 for *P. deserta* and 13 for *P. feae*; Table S3 in the electronic supplementary material). After filtering and interpolation, we obtained a total of 22,376 positions, of which 50.1% and 49.9% were assigned to breeding and non-breeding periods, respectively.

In general, there was substantial variation in timing of migration and in the spatial characteristics of non-breeding distributions among and within species. Based on AIC values, the best-supported models always included species as a fixed effect (Table 3). In addition, year accounted for a small proportion of the total variability in most cases.

**Figure 1:**
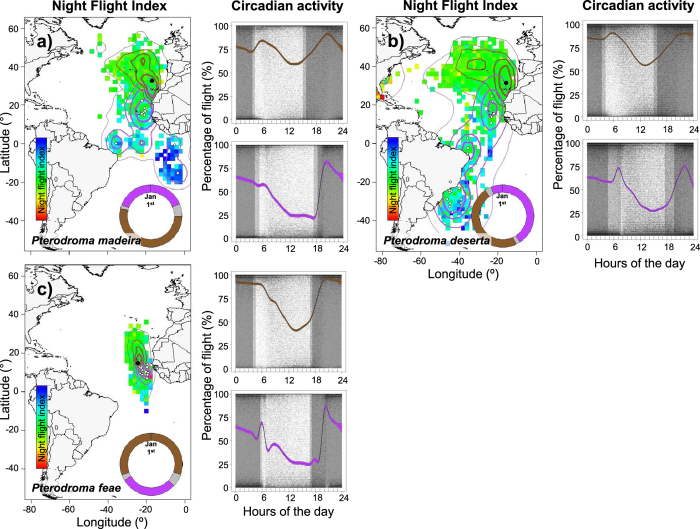
Spatio-temporal variation in the allocation of time spent flying during daylight *vs.* darkness by Macaronesian gadfly petrels, year-round (in a, b and c, for *P. madeira, P. deserta* and *P. feae*, respectively). Night flight index (as grid maps in the left panels) reflects the relative amount of flight allocated to night-time, ranging from blue (*i.e.*, flying exclusively during darkness) to red (*i.e.*, flying exclusively during daylight; see method for details). Specific kernel density distributions (25, 50, 75 and 95%, from thicker to lighter line contours, respectively) are depicted on the respective grid map (in brown for breeding and purple for non-breeding periods). Black circles show the location of the respective breeding colony and white circles represent individual averaged non-breeding positions (computed as averaged coordinates of the 5% UD of each individual) in the appropriate plot. Schematic annual cycle (starting 1^st^ January) is also shown in coloured circles for each species (brown for breeding, purple for non-breeding and grey for migration periods). Circadian flight activity is shown as percentage of daily time spent flying (right-hand panels) separately for breeding and non-breeding periods for each species. The solid lines correspond to the mean for each event estimated using generalized additive mixed models (GAMM) and the coloured regions around the means delimited by dashed lines represent the associated 95% CI of the slopes (brown for breeding and purple for non-breeding periods). Dark grey areas correspond to darkness, and light grey areas to sunrise and sunset periods. Background maps were created using the *maps* and *mapproj* packages of R (R Foundation for Statistical Computing, Vienna, Austria; www.r-project.org) and Adobe Illustrator (Adobe Systems Inc., CA, USA; www.adobe.com/products/illustrator.html).

*P. madeira* arrived at the colony from late February to mid-April, and departed around mid-October to early November, corresponding to a breeding season of 211 ± 26 days. *P. deserta* tended to spent less time around the colony during the breeding season (on average 183 ± 14 days; see also Table S3 in the electronic supplementary material), from late May/mid-June to mid-November/late December. *P. feae* tended to use similar areas during the breeding and non-breeding periods (Fig. 1), and colony arrival and departure often coincided with the equinox periods, thus precluding precise estimation of the timing of breeding for this species. *P. feae* at Cape Verde had the longest breeding season (239 ± 29 days), and timing was different again (from early September/mid-October to mid-April/early June). The distance between the breeding colony to the core non-breeding area (*i.e.*, non-breeding range) was much larger in *P. madeira* and *P. deserta* than in *P. feae* (Table 3). *P. deserta* showed the largest non-breeding range, and also had the longest non-breeding period, whereas *P. feae* showed the smallest range and the shortest period.

The distributions of the tracked birds were concentrated around each colony during the breeding season, although several *P. madeira* and *P. deserta* consistently exploited a large area around the distant Azores archipelago, whereas *P. feae* tended only to use areas around the Cape Verde archipelago (Fig. 1). During the boreal summer, there was a large spatio-temporal overlap between breeding *P. madeira* and *P. deserta* (*ca.* 70.0%), whereas overlap was minimal with non-breeding *P. feae* (*ca.* 1.0%; Table 4). During the boreal winter (when only *P. feae* is breeding), the three species overlapped in a core area in subtropical waters around Cape Verde (Fig. 1 & see Table 4), corresponding to 23%, 56% and 100% of sampled birds for *P. deserta, P. madeira* and *P. feae*, respectively. However, a substantial proportion of *P. deserta* and *P. madeira* (77% and 44%, respectively) migrated further south to different areas between the Equator and the southern Atlantic Ocean during the boreal winter. This included two other distinct areas; off the equatorial coast, or in subtropical waters off Brazil (at 20–40°S; Fig. 1). Most petrels (87%) that migrated further south also staged in the Cape Verde region for several days during their outward and return migrations^30^.

### Habitat modelling

The AUCs obtained with the MaxEnt models were generally larger for the breeding than the non-breeding season (Table 5). The importance of each variable and its contribution to the MaxEnt models differed both between seasons and among species. In jack-knife tests, SST was the most important variable, and also accounted for the highest percentage contribution to breeding, non-breeding and annual models (Table 5). In addition, the WIND variable also tended to be well-ranked in models for the three species. In general, during the breeding season, there was a consistent preference by most birds for areas of warm water (15–23 °C for *P. madeira* and *P. deserta* and 21–27 °C for *P. feae*) and low wind intensity (6–9 m s^−1^ for all species; Fig. S1 in the electronic supplementary material). Similarly, modelling of habitat use during the non-breeding period indicated that birds tended to select areas of even warmer waters (21–28 °C for *P. madeira* and *P. deserta* and 25–28 °C for *P. feae*), and of similar wind intensity (4–9 m s^−1^ for all species; Fig. S1 in the electronic supplementary material). Additionally, suitable non-breeding habitats were predicted for the different taxa using models developed for the same species during the breeding season (Fig. S2 in the electronic supplementary material). The predicted non-breeding distributions of *P. madeira* and *P. deserta* were similar, and indicated that oceanic areas in the South Atlantic should be the most preferred. Such spatial predictions differed from the observed distribution for both taxa. The most suitable areas predicted for *P. feae* concentrated around the Cape Verde archipelago as well as around the Bermuda Islands.

### At-sea activity patterns

**Figure 2:**
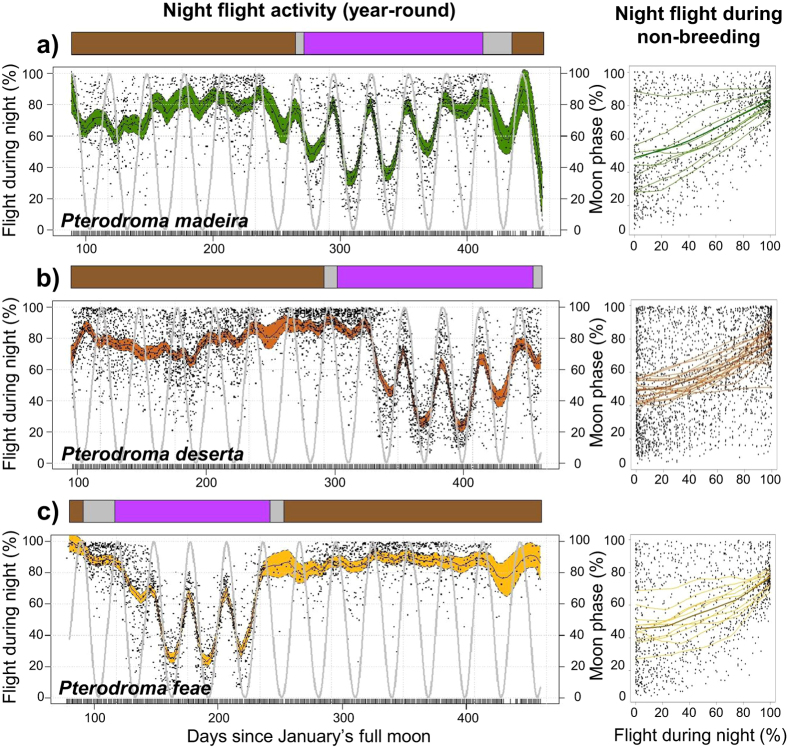
Effect of moonlight on nocturnal flight activity, year-round and during the non-breeding season, for the three Macaronesian gadfly petrels: *P. madeira* in green (**a**) *P. deserta* in brown (**b**) and *P. feae* in yellow (**c**). To appropriately compare different annual cycles under a common lunar cycle, daily percentages of nocturnal time spent flying through the year are re-scaled to the first full moon of every year (*i.e.*, each January’s full moon for the period 2007–2013). The solid lines correspond to the mean for each species estimated using generalized additive mixed models (GAMM), and the coloured regions around the means delimited by dashed lines represent the associated 95% CI of the slopes. Annual lunar cycles are plotted alongside in grey for each species. At the top of each plot, life-cycle events are shown as brown-grey-purple bars (mean of days), representing breeding, individual variability and non-breeding periods, respectively. In addition, percentage of flight time at night during the non-breeding season is shown separately for each species against moonlight (0 represents a new moon, and 100 a full moon). Locally-weighted non-parametric regressions are displayed at individual (thin lines) and specific (thick lines) levels.

Analysis of at-sea activity patterns revealed substantial heterogeneity among species, seasons, non-breeding areas, and daylight and darkness periods, as well as a substantial influence of moonlight, particularly during the non-breeding season (Table S4 in the electronic supplementary material). The three species displayed some similarities in activity patterns, in that birds spent more time flying during the breeding season as well as during the night (Fig. 1 and Table S4). In all three species, flight activity also increased considerably around dawn and dusk during their respective non-breeding seasons (Fig. 1). In addition, nocturnal activity of each species was influenced by moonlight, particularly during the non-breeding season (Fig. 2 and Table S5 in the electronic supplementary material). The proportion of time spent flying clearly fluctuated with moon phase; birds were more active during moonlit nights, and flew less on nights close to new moon phase. This pattern was common to all three species, despite the differences in timing of their annual breeding and migration schedules (Fig. 2).

## Discussion

Until recently, few studies have investigated the migratory ecology of gadfly petrels^10^,^30^,^31^,^32^,^33^. Our study reports for the first time, a comparison of the timing of life-cycle events (breeding and migration), year-round distribution, marine habitat and at-sea activity patterns of three closely-related taxa of gadfly petrels breeding in Macaronesia. By doing so, we also provide relevant information on the ecological divergence of each taxon within this complex of threatened species. These results confirm the small effective population size of each taxon, and highlight their precarious conservation status.

### Spatio-temporal distribution

During the boreal summer (considered here as May-September), breeding *P. deserta* and *P. madeira* exploited a vast oceanic area between the Canaries and Azores^34^, while *P. feae*, unconstrained by breeding duties, were concentrated around the Cape Verde archipelago. Hence, our results indicate that the three taxa are widely distributed in the subtropical waters of the Northern Hemisphere in boreal summertime. In addition, the small populations of *P. deserta* and *P. madeira* prefer to forage at northern latitudes in more temperate waters, largely segregating from *P. feae* around Cape Verde. During the boreal winter (considered here as November-March), *P. feae*, which is breeding, remained around the Cape Verde archipelago. At the same time, a substantial proportion of the tracked *P. deserta* and *P. madeira* spent their entire non-breeding period in the same restricted area used by breeding *P. feae*. This region is influenced by the North Equatorial Current, and its waters are characterized by high sea surface temperatures, low wind speeds and high productivity. Previous studies suggested this is probably an important wintering area for *P. deserta* and *P. madeira*^30^,^32^, and our results confirm this for all three species of Macaronesian gadfly petrels. However, a considerable proportion of the two northern species (77% of *P. deserta* and 44% of *P. madeira*) leapfrogged those birds wintering around Cape Verde, and migrated to equatorial waters, or even further south to a vast area off southern Brazil. Within the tropical and subtropical waters used by *P. deserta* and *P. madeira*, two regions in particular hold a large number of birds: the northern Brazil Current, where both species occurred, and the southern Brazil Current where only *P. deserta* concentrated in high numbers. In addition, a small proportion of *P. madeira* also exploited a vast area in the central Atlantic, between the Equator and the South Atlantic Ocean (around Saint Helena).

Classically, spatio-temporal segregation between related taxa has been seen as a need to avoid conspecific competitors^35^,^36^. Either direct or indirect competition for limited resources certainly implies a cost in terms of reduced foraging efficiency, which would promote segregation in habitat use by different populations in time^37^, space^38^ or diet^39^. In this context, the higher costs of migration assumed by leapfrog migrants (described here for northern breeding birds) has been classically attributed to the avoidance of conspecific competition in non-breeding quarters^40^. However, the small population sizes of the three taxa in our study (both currently and in ancestral times; Table 1)^17^ do not support a competition avoidance hypothesis because we can reasonably assume that the level of resource use in common foraging areas (*e.g.* in Cape Verde waters) is relatively low, and should therefore permit their co-occurrence.

Alternative hypotheses explaining interspecific spatial segregation and leapfrog migration patterns relate to habitat specialization^41^,^42^. For instance, ecological niche models of the northern populations of Bulwer’s petrels (*Bulweria bulwerii*) based on breeding-season data effectively predicted the non-breeding distributions of these same birds in the southernmost part of the non-breeding range^42^. Such results indicated that specific habitat preferences of certain populations may determine the observed distributions and the leapfrog migration described for the species. However, in the present case, habitat modelling incorporating spatially-explicit environmental variables failed to predict the non-breeding distribution of those leapfrog migrants (Fig. S2 in the electronic supplementary material). In addition, at-sea activity patterns of the three species (discussed below in detail) were similar year-round. Outside the breeding season, the nocturnal activity of most birds was synchronised with the moon cycle (the brighter the moon, the higher the flight activity), independently of species or non-breeding region. Therefore, neither competitor avoidance nor habitat specialization (in terms of habitat use and activity patterns) seemed to drive the reported spatial segregation.

Thus, the explanation for the leapfrog distribution described here for the three species of Macaronesian gadfly petrels remains unclear. Our study did not answer the fundamental question of why and how long-distance migrants of *P. deserta* and *P. madeira* trade off the benefit of foraging in equatorial and south Atlantic waters against the energetic cost of flying longer distances. Certainly, hundreds of birds concentrate during summertime in tropical waters around Cape Verde, and only individuals of the northern two species travelled south of the Equator. It seems reasonable then that the higher costs of longer migrations might be compensated by a more efficient exploitation or a higher habitat quality of those more distant wintering grounds, so that neither a short- nor a long-distance migratory strategy would consistently be more successful^43^,^44^. Further ecological studies aiming to disentangle the diet, breeding performance and energetics of those longer-distance migrants (with pre-migration and post-migration assays) would be required to better understand their distribution patterns. Finally, complementary information on distribution and population sizes of ancestral populations, as well as those of previous common ancestors, might help explain the migratory segregation. Although recent molecular studies have suggested that the three extant petrel taxa only diverged recently in Macaronesia (*ca.* 40,000 years ago) and that the most ancestral population would have bred in Madeira (*i.e., P. madeira*)^17^, there is no clear evolutionary scenario that explains the current year-round distribution.

### At-sea behaviour of Macaronesian gadfly petrels

In general, the three Macaronesian gadfly petrels spent more time flying during the breeding than non-breeding periods, indicating more time spent actively searching for prey, which is likely linked to chick provisioning duties. However, it is important to note that this to some extent might be an artefact of the time spent flying over the colony, or inside the burrow, during the chick-rearing period, which is difficult to disentangle from true foraging flight (time spent in the burrow during incubation was removed from our analysis). Regardless, during the non-breeding season, most birds reduced considerably the proportion of time spent in flight at sea, and the bulk of the flight activity was particularly concentrated around sunrise and sunset periods (Fig. 1). As in other seabird species^28^,^31^, an increase in flying activity around sunrise and sunset is assumed to be the response to a higher availability of their prey due to diel vertical migrations (DVM).

The proportion of time spent flying at night at any time of the year was always higher than during daylight (except for a single bird that spent the non-breeding period in the Gulf Stream Current off North America, where the inverse was true; Fig. 1). Nocturnal activity tended to be high and relatively consistent across species while breeding, but such activity varied considerably depending both on non-breeding region and on moon phase. The latter clearly influenced the at-sea behaviour of all species during their respective non-breeding seasons: flight time was higher on moonlit nights (around full moon), and lower on nights with little moonlight (around new moon). Previous studies have also documented the influence of lunar cycles on the night-time activity of seabirds during the non-breeding period^28^,^31^,^45^. However, its influence depends a great deal on the study species: some increase their flight activity on bright moonlit nights^31^,^45^, while others are clearly less active and prefer ‘sit-and-wait’ foraging during such nights^28^,^46^. Interestingly, we report here the same activity patterns in three similar species with asynchronous breeding schedules, and with overlapping and non-overlapping non-breeding areas. Birds from different species behave similarly in different areas and period of the year; for instance, the nocturnal activity of all birds wintering around Cape Verde was synchronised with the moon cycle, regardless of taxon.

Although little is known about the diet of gadfly petrels^21^, the more intense foraging activity during the non-breeding season at twilight and on nights with abundant moonlight, suggests that the species in our study rely on small squid and fish showing DVM^47^,^48^. These diel vertical migrants are largely bathypelagic or mesopelagic during daylight, but they become available to surface predators at night when they come to the surface to feed on macrozooplankton^49^. At twilight (both dawn and dusk) and during nights with intense moonlight, petrels can presumably see more effectively and so are better able to locate and capture prey that are diel vertical migrants^45^. Thus, at least during the non-breeding period, Macaronesian gadfly petrels seem to target prey that use DVM. Finally, the higher flight activity at night might have important conservation implications for these threatened species if it makes them more susceptible to collisions or grounding on fishing boats or other vessels which use powerful lights at night^50^,^51^. Although to the best of our knowledge, none of the gadfly petrel species considered here has been reported dead on a trawler or longliner, the absence of bycatch in fishing gear, collisions with the ship superstructure or contact with oil on-board may just reflect the small populations of the three taxa. Indeed, several gadfly petrel species are known to be disoriented and attracted by artificial light^52^,^53^. All these possibilities require further investigation.

### Phylogeographic and conservation implications

Identifying cryptic and recently-diverged taxa can have important conservation implications^13^. Three non-exclusive intrinsic factors are often considered to contribute to population differentiation in seabirds^4^,^10^: high philopatry, breeding asynchrony and spatio-temporal segregation of foraging areas used year-round. Gadfly petrels are well known for their strong natal philopatry and breeding-site fidelity^10^,^21^,^54^. Indeed, our CMR results confirm this for the three species, indicating that the gadfly petrels tend to return to the same colony in Macaronesia, year after year. Survival rates of the three species, although lower than expected for long-lived petrels^55^,^56^, indicated a high degree of breeding-site fidelity, as adult birds were recurrently recaptured at the same study site. Moreover, despite an extensive ringing program at each site over the last two decades, none of the ringed birds (neither chicks nor adults) was ever recaptured in any other “non-native” or allochthonous site. Thus, in spite of the great mobility, wide distribution, absence of apparent physical barriers to dispersal in these taxa, and the relatively short geographical distance between breeding colonies, the high natal philopatry and breeding-site fidelity seem likely to have reduced gene flow among the ancestral populations and contributed to genetic differentiation^57^,^58^.

Secondly, we present here the first accurate annual schedules for the three taxa thanks to the precise information on timing of breeding, and outward and return migrations of individuals provided by the loggers. These schedules confirm a high degree of breeding allochrony, which might be an adaptation to the more pronounced seasonality of the Madeiran archipelago compared with Cape Verde^59^,^60^. Indeed, if the Madeiran archipelago has been colonised twice by ancestral petrels from Cape Verde^18^,^19^, it is reasonable to think that the phenology of the two colonizing populations adapted independently to the new environmental scenario. The observation that the breeding schedules of *P. deserta* and *P. madeira* differ only slightly, but are distinct from that of *P. feae* seems to support the hypothesis of adaptation to local environmental conditions, which could therefore have played an important role in the differentiation of their ancestral populations^37^.

Finally, population differentiation might also arise from divergence in foraging distribution, habitat use or behaviour during the breeding or non-breeding periods^10^,^61^. In our case, although the three species shared common foraging areas and strategies, some non-breeding grounds were used to a large extent by a single species. Thus, as found in other seabird species complexes^10^, the combination of factors outlined above did likely restrict gene flow and presumably drove the population differentiation of the three Macaronesian gadfly petrel taxa.

Overall, our study has provided new and complementary insights into spatial ecology, ecological similarity and divergence, and taxonomy of these little-known gadfly petrel species.

Our findings confirm the existence of three distinct evolutionary significant units^17^,^19^, which certainly implies smaller effective population sizes for each taxon at the global scale. All this information highlights the already delicate conservation status of such birds, and underlines the need for greater conservation efforts at both breeding and non-breeding sites.

## Additional Information

**How to cite this article**: Ramos, R. *et al*. Global spatial ecology of three closely-related gadfly petrels. *Sci. Rep.* **6**, 23447; doi: 10.1038/srep23447 (2016).

## Supplementary Material

Supplementary Information

## Figures and Tables

**Table 1:** Gadfly petrels breeding in the Macaronesian archipelagos in the North Atlantic Ocean.

**Species**	**Breeding distribution**	**Sampled locality**	**Longitude (°)**	**Latitude (°)**	**Estimated global population size**	**Conservation status (IUCN 3.1)**	**Breeding season**	**Reference**
**Zino’s petrel** (Madeira petrel) *Pterodroma madeira*	Madeira Island	Madeira highlands	−16.94	32.76	65–80	Critically endangered	April-Sept	Zino *et al*. 2011
**Desertas petrel** (Bugio petrel) *Pterodroma deserta*	Desertas Islands (Madeira)	Bugio Islet	−16.40	32.48	160–180	Endangered	June-Dec	Oliveira *et al*. 2008
**Cape Verde petrel** (Gon-gon) *Pterodroma feae*	Cape Verde archipelago	Fogo Island	−24.34	14.95	500	Near threatened	Nov-May	Ratcliffe *et al*. 2000

Study colony characteristics.

**Table 2:** Estimated recapture and survival probabilities based on Capture-Mark-Recapture models for the three Macaronesian gadfly petrels (Zino’s petrel: *P. madeira*, Desertas petrel: *P. deserta* and Cape Verde petrel: *P. feae*; see in the electronic supplementary material for details).

	***P. madeira***	***P. deserta***	***P. feae***
Capture probability (mean ± SE)			
2007	0.50 ± 0.15	0.67 ± 0.27	–
2008	0.25 ± 0.15	0.55 ± 0.10	0.07 ± 0.06
2009	0.67 ± 0.18	0.19 ± 0.05	0.07 ± 0.05
2010	0.56 ± 0.11	0.11 ± 0.04	0.48 ± 0.09
2011	0.42 ± 0.09	0.13 ± 0.05	0.22 ± 0.07
2012	0.41 ± 0.11	0.44 ± 0.08	0.35 ± 0.07
2013	–	0.51 ± 0.09	0.26 ± 0.07
2014	–	0.54 ± 0.10	0.43 ± 0.11
Survival probability (mean ± SE)			
	0.83 ± 0.05	0.75 ± 0.03	0.80 ± 0.04
Total of ringed adults	64	217	128
Total of ringed chicks	155	170	6
Recaptures of adults ringed as chicks	6	8	1

Additionally, the main figures of the ringing results are provided by each taxon as: Total of ringed adults, Total of ringed chicks and Recaptures of adults ringed as chicks. Notably, no recapture of “non-native” birds is reported in any of the studied sites in spite of the extensive ringing program at each breeding colony over the last two decades.

**Table 3:** Generalised linear mixed models (GLMMs) testing for species effect on five migration characteristics of Macaronesian gadfly petrels.

	**Colony departure date**	**Colony arrival date**	**Duration of the non-breeding period (days)**	**Distance between colony and non-breeding area (km)**	**Centroid latitude during the non-breeding period (°)**
(a)					
Species	**320.3**	**305.7**	**343.3**	**655.7**	**321.0**
Constant	457.9	433.1	384.2	712.8	337.7
(b)					
Fixed effects (estimate ± SE)					
* P. feae* (Intercept)	126.9 ± 6.2	248.1 ± 3.7	125.1 ± 6.2	740.2 ± 535.5	10.2 ± 4.5
* P. madeira*	161.1 ± 7.2	−168.3 ± 5.9	28.8 ± 10.0	2287.0 ± 771.4	−3.7 ± 7.4
* P. deserta*	206.4 ± 6.4	−95.2 ± 4.8	56.9 ± 8.1	4576.7 ± 654.5	−18.9 ± 5.9
Random effect (variance ± SE)					
Year	123.5 ± 11.1	0.0 ± 0.0	0.0 ± 0.0	417930.0 ± 646.5	0.1 ± 0.1
Residual	216.0 ± 14.7	175.3 ± 13.24	498.5 ± 22.3	2691877.0 ± 1640.7	268.5 ± 16.4

(a) Results of Akaike’s Information Criterion (AIC) analysis for the two competing models: with and without species factor. Values refer to AIC adjusted for small sample sizes (AICc). The best-supported model (in bold) included in all the five cases species as a fixed effect. (b) Parameter estimates ( ±standard error) from species-dependent GLMMs. All evaluated models included year of sampling as random effect.

**Table 4:** Spatial overlap in the 95% kernel UD of Macaronesian gadfly petrels tracked during their respective breeding (breeding) and non-breeding (non-breeding) periods.

	***P. madeira* breeding**	***P. deserta* breed breeding**	***P. feae* non-breeding**	***P. madeira* non-breeding**	***P. deserta* non-breeding**
*P. deserta* breeding	**69.8**				
*P. feae* non-breeding	**0.8**	**0.9**			
*P. madeira* non-breeding	14.0	15.3	21.0		
*P. deserta* non-breeding	15.6	17.8	5.7	**35.9**	
*P. feae* breeding	14.0	12.8	16.6	**44.7**	**17.1**

Spatio-temporal overlap events are depicted in bold.

**Table 5:** Estimates of model fit and relative importance (percent contribution) of the environmental variables to the probability of occurrence of each species of petrel (values over 15% are marked bold).

**Species**	**Period**	**Season**	**Percent contribution**						**Permutation importance**				
			**AUC**	**BAT**	**BATG**	**CHLa**	**SST**	**WIND**	**BAT**	**BATG**	**CHLa**	**SST**	**WIND**
*P. madeira*	May-Sept	Breeding	0.966	8.5	8.4	7.2	**48.5**	**27.5**	4.0	4.8	4.9	**52.4**	**33.8**
	Nov-Mar	Non-breeding	0.933	14.9	3.7	**20.1**	**44.7**	**16.6**	**22.7**	3.4	**17.9**	**40.2**	**15.8**
	Year round		0.880	10.0	6.0	**22.5**	**50.9**	10.7	12.2	8.0	**25.0**	**38.7**	**16.1**
*P. deserta*	May-Sept	Breeding	0.951	6.7	8.0	3.3	**55.9**	**26.1**	6.2	6.0	2.5	**57.2**	**28.2**
	Nov-Mar	Non-breeding	0.886	12.0	5.3	5.8	**43.2**	**33.7**	14.2	6.0	9.9	**38.5**	**31.4**
	Year round		0.809	2.5	3.3	12.8	**74.7**	6.7	5.1	5.9	12.3	**64.7**	11.9
*P. feae*	Nov-Mar	Breeding	0.990	10.7	4.8	**16.5**	**52.2**	**15.8**	9.4	0.5	11.7	**66.9**	11.6
	May-Sept	Non-breeding	0.987	14.1	1.4	**20.9**	**33.7**	**29.9**	6.0	1.0	12.4	**27.4**	**53.2**
	Year round		0.944	9.0	5.9	**21.0**	**58.3**	5.8	5.5	3.1	**17.4**	**71.7**	2.3
Total	Year round		0.805	5.3	2.5	13.6	**74.2**	4.3	8.3	5.3	**18.6**	**59.2**	8.6

Separate models were built for each breeding and non-breeding period, and year-round (see the supplementary material for details). Values shown are averages over 100 model replicates. AUC: area under the receiver operating characteristic curve; BAT: bathymetry; BATG: gradient of BAT; CHLa: chlorophyll a concentration; SST: sea surface temperature; WIND: wind speed.
