# Supplementary material for: Global spatial ecology of three closely-related gadfly petrels
Source: Sci Rep. 2016 Mar 22;6:23447. doi: 10.1038/srep23447 (PMC4802315; doi:10.1038/srep23447)
Supplement: Supplementary Information [file srep23447-s1.doc]

**Global spatial ecology of three closely-related gadfly petrels**

Raül Ramos, Iván Ramírez, Vitor H. Paiva, Teresa Militão, Manuel Biscoito, Dília Menezes, Richard A. Phillips, Francis Zino, Jacob González-Solís

**Population dynamics and demographic analysis.** Demographic parameters were estimated with CMR models ^1^, using M-Surge version 1.8 ^2^ and a total of 64, 217 and 128 adult capture-recapture histories (*i.e.*, individuals) over the periods of 2006-2012, 2006-2014 and 2007-2014 for *P. madeira*, *P. deserta* and *P. feae*, respectively. We started with the Cormack-Jolly-Seber (CJS) model where survival (Φ, probability that a petrel alive at year *t* survives until year *t*+1) and capture (p, probability that a petrel alive and present at the breeding colony at year *t* is caught during the year *t*) were time dependent (*t*). The fit of the general model to the data was investigated with goodness-of-fit (GOF) tests for each species using program U-Care version 2.2 (Table S1) ^3^. Model selection was based on the Akaike Information Criterion corrected for small sample size and over-dispersion (QAICc) ^4^. When comparing two models, if ΔQAICc > 2, the preferred model is the one with the smallest QAICc value (*i.e.* the most parsimonious model in terms of the number of parameters and model deviance) ^1^. First, models with various capture probability structures were compared, and then we considered models with various survival probability structures (Table S2).

**Tracking data, filtering and its associated modelling.** Two positions per day (local midday and midnight) were estimated from the light data using the BASTrak software suite ^5^, with an average error of ~200 km (or ~ 2°) ^6^. We estimated dawn and dusk times by inspecting the integrity of each light curve; latitude was derived from daylength, and longitude from the time of local midday with respect to Greenwich Mean Time. We excluded long periods spent in burrows during incubation, based on light data recorded by the logger. To filter unrealistic positions, we removed those that were (1) obtained from light curves showing interference at dawn or dusk; (2) within the 20 closest days to the equinoxes; and (3) that resulted in unrealistic flight speeds (>40 km h^-1^ sustained over 48 h) using user-defined routines written in R ^7^. Filtered data were smoothed twice by interpolating intermediate fixes between successive locations with fixed start and end points around any periods of missing data ^6^. Timing of departure and arrival of individual birds at the different breeding areas was determined visually: departure date was considered to be the first day when the bird’s location was outside the cluster of positions of the previous days that corresponded to the breeding area, followed by directed movement away from this area; and arrival date was considered to be the first day of return to the breeding region after a directed movement towards that area (Table S3). We estimated five phenological and spatial parameters for every complete migration cycle (Table S3): (1) departure date, (2) arrival date, (3) duration of the non-breeding period (in days), (4) non-breeding range (orthometric distance between the breeding colony and the average of all locations within the 5% Utilization Distribution [UD]; in km), and (5) latitude of the centroid of the core of the non-breeding distribution (mean latitude of all positions within the 5% UD; in degrees). We used Q-Q plots to check for normality of the distributions of these variables (by species). We evaluated the effect of species on these non-breeding parameters using generalized linear mixed models (GLMMs). Each of the five parameters described above was the response variable, species was the explanatory variable (fixed effect), and year of sampling was a random effect in the models (Table 3). Gaussian distribution of error terms and a log-link function were used in the modelling. For the purposes of model comparison, each pair of nested models were fitted using maximum likelihood ^8,9^. The best-supported models were selected using the Akaike Information Criteria corrected for small sample sizes (AICc) and the corresponding AICc weights; models were deemed strongly supported if they differed from the best model by up to two AICc units ^8^. GLMMs were conducted in R version 2.8.1 (R Development Core Team 2010) with additional functions provided by the R packages *lme4* (glmer) ^10^ and *MuMIn* (dredge) ^11^.

The loggers also tested for immersion in sea water every 3 s using 2 electrodes, and provided a value (0 to 200) corresponding to the sum of positive tests in each 10-min period, which can be transformed to the proportion of time spent wet, *i.e.*, when the bird was sitting on the sea surface or diving. Light and immersion data were used simultaneously to (1) distinguish time spent at sea from time at the colony (darkness during daylight indicates the bird is in the burrow), and (2) estimate the percentage of time spent flying as a proxy of foraging effort ^12^, separately for daylight and darkness periods. As daily activity budgets vary seasonally in many seabird species, we modelled the dynamics of time spent flying throughout the annual cycle in the three species using GLMMs with binomial error structure. Specifically, we first evaluated the associations between species, annual season and daylight (*i.e.*, day or night) factors with the percentage of time spent flying (Table S4). Secondly, we also evaluated the effect of moonlight on the flight activity throughout the annual cycle in the three species, separately (Table S5). In this case, we ran three independent sets of GLMMs, one for each species, to provide clear biological information on the flight activity pattern of each species. As previously explained, the best-supported models were selected on the basis of AICc values and their corresponding AICc weights ^8^. Complementarily, for plotting purposes, activity budgets were also modelled using generalized additive mixed models (GAMMs). We used the library *mgcv* in R, based on penalized regression splines and generalized cross-validation to select the appropriate smoothing parameters ^13^. GAMMs combine the utilities of linear mixed models ^14^ and generalized additive models ^15^ so that random factors, fixed factors and nonlinear predictor variables can all be estimated in the same statistical model. Specifically, we included species as a fixed factor, the number of days since January’s full moon of each year as a smooth term, and bird identity as a random term (Fig. 2). This allowed us to determine cyclicity in the time spent flying over the year for each species, particularly in relation to the lunar cycle and the life-history stage (breeding, migrating, wintering). Finally, percentage of time spent flying at night during the non-breeding season was also regressed against moonlight levels (from 0 during a new moon, to 100 during a full moon) at individual and population levels, using locally-weighted non-parametric regressions (Fig. 2) ^16^.

**Environmental data and habitat modelling.** To determine the oceanographic characteristics of habitats used by the tracked birds, we considered the seafloor depth (BAT, m), surface chlorophyll *a* concentration (CHLa, mg m^−3^), salinity (SAL, gr of salt per 1000 gr of water), sea surface temperature (SST, °C), and wind speed (WIND, m s^−1^). All remote sensing products were extracted from NOAA CoastWatch ([coastwatch.pfeg.noaa.gov/](http://coastwatch.pfeg.noaa.gov/)). The static BAT variable and monthly composites of CHLa, SST and WIND (dynamic variables downloaded for the period 2007-2013) were rescaled to a common spatial resolution of 2°, which matches the accuracy of geolocation data. Gradients for BAT, CHLa and SST were also considered (BATG, CHLG and SSTG, respectively). For every annual cycle, values for dynamic variables were the averages for the breeding and non-breeding periods, or year-round for each species, as appropriate. To exclude redundant variables, pair-wise correlations among the eight environmental variables were evaluated separately for each period (*i.e.* breeding, non-breeding, and year-round) using Spearman methodology with Holm adjustments (Table S6). Gadfly petrel habitat suitability models were developed using the MaxEnt v.3.3.3e software ^17^, a program for modelling ecological niches from presence-only species records. Habitat models were run with six non-redundant variables (BAT, BATG, CHLa, SST, SSTG and WIND) for each of the ten data subsets, including specific breeding (3 models), non-breeding (3 models) and year-round subsets (3 models), plus a supra-specific global subset (1 models). All models of habitat use were developed on the basis of a logistic output format, and with 100 bootstrapped replicates, each built using randomly-sampled subsets of 10% of the bird positions as training points. This conservative approach (90% of the seabird records were used for model testing) avoids model over-fitting and minimizes effects of spatial autocorrelation on both seabird presence and environmental covariates. The data were jack-knifed to evaluate the importance of each variable in explaining the observed distribution. The percent contribution of each variable was calculated on the basis of the increase in the regularized model gain averaged over each model run. To determine the permutation importance of each variable, the values in the training presence and background data were varied randomly, and the resulting change in the area under the curve (AUC) statistic was examined, normalized to percentages ^17^. The results were summarized as the average of the 100 models, and model evaluation was performed using the AUC statistic, which measures the ability of model predictions to discriminate seabird presence from background points (Table 5). In the results, all means are presented ± standard deviation, unless otherwise stated.

**Table S1.** Results of goodness-of-fit (GOF) tests of Cormack-Jolly Seber model (CJS), including time-dependent parameters (Φ *_t_* p *_t_*) for birds sampled in Madeira (*P. madeira*), Desertas (*P. deserta*) and Cape Verde (*P. feae*) archipelagos between 2006-2012, 2006-2014 and 2007-2014, respectively for each breeding site.

|  | Test 3SR | | |  | Test 3SM | | |  | Test 2CT | | |  | Test 2CL | | |  | Sum of Tests | | |
| --- | --- | --- | --- | --- | --- | --- | --- | --- | --- | --- | --- | --- | --- | --- | --- | --- | --- | --- | --- |
|  | df | χ^2^ | *P* |  | df | χ^2^ | *P* |  | df | χ^2^ | *P* |  | df | χ^2^ | *P* |  | df | χ^2^ | *P* |
| *P. madeira* | 5 | 0.0 | 1.000 |  | 3 | 0.0 | 1.000 |  | 2 | 1.4 | 0.494 |  | 3 | 0.9 | 0.626 |  | 12 | 2.3 | 1.000 |
| *P. deserta* | 7 | 5.2 | 0.631 |  | 6 | 2.2 | 0.898 |  | 6 | 3.0 | 0.815 |  | 4 | 4.5 | 0.339 |  | 12 | 15.0 | 0.900 |
| *P. feae* | 6 | 3.1 | 0.796 |  | 5 | 4.1 | 0.535 |  | 5 | 3.7 | 0.588 |  | 4 | 4.2 | 0.382 |  | 20 | 15.1 | 0.770 |

Tests 3 (3SR and 3SM) check the homogeneity of recapture histories while tests 2 (2CT and 2CL) examine the independence between last release and next recapture (Burnham & Anderson 1998); df degrees of freedom; χ^2^ Pearson's chi-squared statistic; *P* significance of the χ^2^ test.

**Table S2**. Modelling capture (p) and survival (Φ) probabilities of the adult gadfly petrels sampled in Madeira (*P. madeira*), Desertas (*P. deserta*) and Cape Verde (*P. feae*) archipelagos.

| *nº* | *model* | *np* | *DEV* | *QAIC* | *ΔQAIC* |
| --- | --- | --- | --- | --- | --- |
| *P. madeira* | |  |  |  |  |
| *Modelling capture probability (p)* | |  |  |  |  |
| 1 | Φ (*t*) p (*t)* | 15 | 151.6 | 173.6 | 5.0 |
| 2 | Φ (*t*) p (·*)* | 11 | 182.9 | 204.9 | 36.3 |
| *Modelling survival probability (*Φ*)* | |  |  |  |  |
| **3** | **Φ (*·*) p (*t)*** | **9** | **138.6** | **168.6** | **0.0** |
| *P. deserta* | |  |  |  |  |
| *Modelling capture probability (p)* | |  |  |  |  |
| 1 | Φ (*t*) p (*t)* | 15 | 635.0 | 65.0 | 8.2 |
| 2 | Φ (*t*) p (·*)* | 9 | 667.7 | 685.7 | 28.9 |
| *Modelling survival probability (*Φ*)* | |  |  |  |  |
| **3** | **Φ (*·*) p (*t)*** | **9** | **638.8** | **656.8** | **0.0** |
| *P. feae* | |  |  |  |  |
| *Modelling capture probability (p)* | |  |  |  |  |
| 1 | Φ (*t*) p (*t)* | 13 | 368.8 | 394.8 | 3.0 |
| 2 | Φ (*t*) p (·*)* | 8 | 385.7 | 401.7 | 10.0 |
| *Modelling survival probability (*Φ*)* | |  |  |  |  |
| **3** | **Φ (*·*) p (*t)*** | **8** | **375.8** | **391.8** | **0.0** |

np number of parameters estimated; DEV deviance; QAICc quasi-likelihood Akaike’s information criterion values; ΔQAIC difference between the current and the lowest QAICc model; t time; (·) constant; * interaction. In bold characters denoted the models considered.

**Table S3.** Migration characteristics (mean ± SD, and range in parentheses) of Macaronesian gadfly petrels from the North Atlantic Ocean. For each taxon, “Total” refers to total number of migrations tracked.

| Species | Year | n | Colony  departure date | Colony  arrival date | Duration of the non- breeding period (days) | Distance between colony and  non-breeding area (km) | Centroid latitude during  the non-breeding period (°) |  |
| --- | --- | --- | --- | --- | --- | --- | --- | --- |
| *P. madeira* | 2007 | 2 | 13 Oct ± 0.7  (13 Oct|14 Oct) | 28 Feb ± 4.9  (25 Feb|04 Mar) | 137.5 ± 0.7  (137|138) | 2877.3 ± 1751.2  (1639.0|4115.6) | 9.4 ± 12.9  (0.2|18.5) | |
|  | 2008 | 3 | 21 Oct ± 10.1  (12 Oct|01 Nov) | 25 Mar ± 24.8  (25 Feb|09 Apr) | 155.7 ± 34.5  (116|179) | 2524.9 ± 1208.2  (1787.7|3919.3) | 10.9 ± 10.0  (-0.6|17.0) | |
|  | 2009 | 3 | 17 Oct ± 4.9  (12 Oct|21 Oct) | 29 Mar ± 26.3  (28 Feb|19 Apr) | 163.0 ± 28.6  (130|181) | 3734.8 ± 1756.3  (2027.2|5536.1) | 0.0 ± 15.1  (-15.3|14.9) | |
|  | **Total** | **8** | **17 Oct ± 7.0**  **(12 Oct|01 Nov)** | **20 Mar ± 23.3**  **(25 Feb|19 Apr)** | **153.9 ± 26.2**  **(116|181)** | **3066.7 ± 1436.6**  **(1639.0|5536.1)** | **6.4 ± 12.1**  **(-15.3|18.5)** | |
| *P. deserta* | 2008 | 5 | 01 Dec ± 10.8  (16 Nov|12 Dec) | 08 Jun ± 9.7  (27 May|18 Jun) | 188.6 ± 18.9  (166|214) | 4643.1 ± 1965.5  (1975.9|7515.9) | -5.2 ± 15.8  (-28.8|15.5) | |
|  | 2009 | 7 | 01 Dec ± 16.4  (12 Nov|31 Dec) | 30 May ± 7.1  (23 May|11 Jun) | 180.4 ± 12  (162|194) | 6216.8 ± 2347.2  (1092.8|7674.5) | -12.2 ± 28.6  (-33.1|33.6) | |
|  | 2010 | 2 | 06 Dec ± 7.1  (01 Dec|11 Dec) | 24 May ± 0.7  (24 May|25 May) | 169.5 ± 6.4  (165|174) | 7550.9 ± 424.2  (7250.9|7850.8) | -30.8 ± 2.6  (-32.6|-29.0) | |
|  | 2011 | 4 | 01 Dec ± 12.3  (13 Nov|10 Dec) | 01 Jun ± 5.2  (28 May|08 Jun) | 182.8 ± 13.3  (173|202) | 3583.7 ± 2242.5  (1356.4|6565.1) | 3.8 ± 17.6  (-19.9|21.7) | |
|  | **Total** | **18** | **01 Dec ± 12.4**  **(12 Nov|31 Dec)** | **01 Jun ± 8.1**  **(23 May|18 Jun)** | **182.0 ± 14.2**  **(162|214)** | **5342.8 ± 2347.7**  **(1092.8|7850.8)** | **-8.8 ± 22.5**  **(-33.1|33.6)** | |
| *P. feae* | 2007 | 3 | 08 May ± 18.0  (18 Apr|23 May) | 15 Sep ± 22.5  (02 Sep|11 Oct) | 130.0 ± 40.1  (102|176) | 458.4 ± 152.8  (282.6|559.1) | 13.3 ± 0.8  (12.8|14.2) | |
|  | 2008 | 2 | 09 May ± 30.4  (18 Apr|31 May) | 08 Sep ± 9.9  (01 Sep|15 Sep) | 114.5 ± 30.4  (93|136) | 786.4 ± 111.6  (707.5|865.3) | 9.1 ± 0.1  (9.0|9.1) | |
|  | 2009 | 2 | 30 May ± 26.9  (11 May|18 Jun) | 10 Sep ± 11.3  (02 Sep|18 Sep) | 95.0 ± 26.9  (76|114) | 799.0 ± 359.0  (545.1|1052.8) | 8.6 ± 2.7  (6.7|10.5) | |
|  | 2011 | 1 | 13 Apr | 02 Sep | 142 | 870.2 | 8.8 | |
|  | 2012 | 4 | 09 Apr ± 10.0  (02 Apr|19 Apr) | 09 Sep ± 18.0  (02 Sep|28 Sep) | 145.0 ± 9.2  (133|156) | 701.3 ± 219.3  (494.2|1008.2) | 9.5 ± 1.7  (7.1|11.1) | |
|  | 2013 | 1 | 31 May | 03 Sep | 95 | 798.8 | 9.5 | |
|  | **Total** | **13** | **02 May ± 24.6**  **(09 Apr|18 Jun)** | **04 Sep ± 10.8**  **(01 Sep|11 Oct)** | **125.1 ± 28.5**  **(76|176)** | **693.9 ± 219.9**  **(282.6|1052.8)** | **10.1 ± 2.2**  **(6.7|14.2)** | |

**Table S4.** Generalised linear mixed models (GLMMs) testing for potential effects on daily activity budget of Macaronesian gadfly petrels along the annual cycle (estimated as percentage of time spent flying). (a) Candidate models evaluated to fit the data corresponding to daily activity budget and their associated measures of information (AICc: corrected Akaike’s Information Criterion; ΔAICc: AICc increments and AICc Wgt: AICc weights). The most complete model included species, annual season and daylight (*i.e.*, day or night) factors, as well as species×season and season×daynight interactions. All evaluated models included individual identity as a random factor. The best-supported model is shown in bold. (b) Parameter estimates (±SE) from the best-supported model, and their significance.

|  |  | Time-activity budget | | |
| --- | --- | --- | --- | --- |
| a) | *k* | *AICc* | *ΔAICc* | *AICc Wgt* |
| **species + season + daynight + species×season + season×daynight** | **9** | **19473.8** | **0.00** | **0.99** |
| species + season + daynight + season×daynight | 7 | 19483.0 | 9.27 | 0.01 |
| species + season + daynight + species×season | 8 | 19493.6 | 19.80 | 0.00 |
| species + season + daynight | 6 | 19503.8 | 30.00 | 0.00 |
| species + season | 5 | 21323.5 | 1849.71 | 0.00 |
| species + daynight | 5 | 24528.7 | 5054.94 | 0.00 |
| season + daynight | 4 | 19543.1 | 69.30 | 0.00 |
| species | 4 | 25947.7 | 6473.96 | 0.00 |
| season | 3 | 21361.5 | 1887.69 | 0.00 |
| daynight | 3 | 24529.3 | 5055.53 | 0.00 |
| constant | 2 | 25948.3 | 6474.55 | 0.00 |
| b) |  |  |  |  |
| *Fixed effects* |  | *Estimate ± SE* | *z* | *p* |
| *P. feae* & breeding & daylight (Intercept) | | 1.44±0.06 | 22.78 | <0.001 |
| *P. deserta* |  | 0.40±0.08 | 5.16 | <0.001 |
| *P. madeira* |  | -0.14±0.08 | -1.72 | 0.085 |
| Non-breeding |  | -2.62±0.08 | -32.05 | <0.001 |
| Night |  | 1.21±0.07 | 17.24 | <0.001 |
| *P. deserta* × Non-breeding |  | -0.17±0.09 | -1.81 | 0.070 |
| *P. madeira* × Non-breeding |  | 0.16±0.10 | 1.53 | 0.125 |
| Non-breeding × night |  | 0.40±0.08 | 4.80 | <0.001 |
| *Random effect* |  | *Variance ± SD* | | |
| Individual |  | 0.01±0.01 | | |

**Table S5. Generalised linear mixed models (GLMMs) testing for potential effects on daily activity budget of Macaronesian gadfly petrels along the annual cycle (estimated as percentage of time spent flying). (a) Candidate models evaluated to fit the data corresponding to daily activity budget and their associated measures of information (AICc: corrected Akaike’s Information Criterion; ΔAICc: AICc increments and AICc Wgt: AICc weights). The most complete model included species, annual season and daylight (*i.e.*, day or night) factors, as well as species×season and season×daynight interactions. All evaluated models included individual identity as a random factor. The best-supported model is shown in bold. (b) Parameter estimates (±SE) from the best-supported models, and their significance.**

| a) |  | *P. madeira* | | |  | *P. deserta* | | |  | *P. feae* | | |
| --- | --- | --- | --- | --- | --- | --- | --- | --- | --- | --- | --- | --- |
|  | *k* | *AICc* | *ΔAICc* | *AICc Wgt* |  | *AICc* | *ΔAICc* | *AICc Wgt* |  | *AICc* | *ΔAICc* | *AICc Wgt* |
| **season + moon + season×moon** | **5** | **5253.4** | **0.0** | **1.0** |  | **10577.1** | **0.0** | **1.0** |  | **5031.6** | **0.0** | **1.0** |
| season + moon | 4 | 5270.6 | 17.2 | 0.0 |  | 10602.4 | 25.3 | 0.0 |  | 5058.7 | 27.11 | 0.0 |
| season | 3 | 5385.8 | 132.5 | 0.0 |  | 10748.2 | 171.1 | 0.0 |  | 5124.7 | 93.1 | 0.0 |
| moon | 3 | 6310.2 | 1056.8 | 0.0 |  | 13051.9 | 2474.8 | 0.0 |  | 6232.7 | 1201.1 | 0.0 |
| constant | 2 | 6437.0 | 1183.6 | 0.0 |  | 13215.7 | 2638.7 | 0.0 |  | 6299.0 | 1267.4 | 0.0 |
|  |  |  | | |  |  | | |  |  | | |
| b) |  | *P. madeira* | | |  | *P. deserta* | | |  | *P. feae* | | |
| *Fixed effects* |  | *Estimate ± SE* | *z* | *p* |  | *Estimate ± SE* | *z* | *p* |  | Estimate ± SE | z | p |
| Breeding (Intercept) |  | 1.60±0.12 | 13.08 | <0.001 |  | 2.21±0.08 | 26.07 | <0.001 |  | 2.00±0.14 | 14.05 | <0.001 |
| Non-breeding |  | -2.61±0.12 | -21.01 | <0.001 |  | -2.82±0.10 | -29.09 | <0.001 |  | -2.92±0.13 | -22.10 | <0.001 |
| Moon |  | 0.47±0.16 | 2.96 | 0.003 |  | 0.19±0.14 | 1.38 | 0.169 |  | 0.08±0.17 | 0.45 | 0.657 |
| Non-breeding × moon |  | 0.89±0.20 | 4.39 | <0.001 |  | 0.83±0.16 | 5.23 | <0.001 |  | 1.13±0.21 | 5.39 | <0.001 |
| *Random effect* |  | *Variance ± SD* | | |  | *Variance ± SD* | | |  | Variance ± SD | | |
| Individual |  | 0.07±0.26 | | |  | 0.02±0.10 | | |  | 0.08±0.28 | | |

**Table S6.** Analysis of collinearity between the five environmental variables used in the habitat models (breeding: April to August, non-breeding: October to February of consecutive years, and year-round: January to December). For each combination, the matrices show the sign and magnitude of the Spearman correlation coefficient (above diagonal) and the significance level (*P*-values; below diagonal). Highly correlated (-rs- > 0.5) predictors depicted in bold. BAT: bathymetry, BATG: bathymetry gradient, CHLa: chlorophyll *a* concentration, CHLG: CHLa gradient, SAL: salinity, SST: sea surface temperature, SSTG: SST gradient, WIND: wind speed.

| May-Sept | **BAT** | **BATG** | **CHLa** | CHLG | SAL | **SST** | SSTG | WIND |
| --- | --- | --- | --- | --- | --- | --- | --- | --- |
| **BAT** | ***** | -0.153 | 0.445 | 0.468 | -0.195 | -0.041 | 0.275 | -0.132 |
| **BATG** | <0.001 | ***** | -0.083 | -0.020 | 0.011 | 0.077 | -0.070 | -0.043 |
| **CHLa** | <0.001 | <0.001 | ***** | **0.905** | **-0.514** | -0.320 | **0.577** | 0.185 |
| CHLG | <0.001 | 0.733 | <0.001 | ***** | -0.419 | -0.125 | 0.497 | 0.022 |
| SAL | <0.001 | 0.733 | <0.001 | <0.001 | ***** | **0.669** | -0.364 | **-0.580** |
| **SST** | 0.109 | <0.001 | <0.001 | <0.001 | <0.001 | ***** | -0.359 | **-0.795** |
| SSTG | <0.001 | <0.001 | <0.001 | <0.001 | <0.001 | <0.001 | ***** | 0.230 |
| WIND | <0.001 | 0.109 | <0.001 | 0.733 | <0.001 | <0.001 | <0.001 | ***** |
|  |  |  |  |  |  |  |  |  |
|  |  |  |  |  |  |  |  |  |
| Nov-Mar | **BAT** | **BATG** | **CHLa** | CHLG | SAL | **SST** | SSTG | WIND |
| **BAT** | ***** | -0.153 | 0.445 | 0.458 | -0.236 | -0.154 | 0.224 | 0.031 |
| **BATG** | <0.001 | ***** | 0.000 | 0.040 | 0.026 | 0.089 | -0.015 | -0.002 |
| **CHLa** | <0.001 | >0.999 | ***** | **0.937** | **-0.598** | -0.484 | **0.645** | 0.439 |
| CHLG | <0.001 | 0.164 | <0.001 | ***** | **-0.586** | -0.368 | **0.578** | 0.353 |
| SAL | <0.001 | 0.695 | <0.001 | <0.001 | ***** | **0.579** | -0.396 | -0.408 |
| **SST** | <0.001 | <0.001 | <0.001 | <0.001 | <0.001 | ***** | -0.450 | **-0.789** |
| SSTG | <0.001 | >0.999 | <0.001 | <0.001 | <0.001 | <0.001 | ***** | 0.376 |
| WIND | 0.474 | >0.999 | <0.001 | <0.001 | <0.001 | <0.001 | <0.001 | ***** |

**Figure S1.** Habitat suitability of Macaronesian gadfly petrels for breeding and non-breeding periods assessed from MaxEnt models (in a and b for *P. madeira*, in c and d for *P. deserta* and in e and f for *P. feae*). Response curves from the habitat models showing relationship of presence probability and the two most influencing environmental variables (sea surface temperature [SST] and wind intensity [WIND]) for breeding and non-breeding seasons. The curves show how the logistic prediction of each variable changes, keeping all other environmental variables at their average sample value. Red lines show the mean bootstrap value of 100 iterations, and the blue shade represents the mean +/- one standard deviation. Maps display for each taxa the habitat modelling performed with the breeding and non-breeding positions separately and with the environmental conditions during the respective period. Kernel density distributions (25, 50, 75 and 95%, from thicker to lighter line contours, respectively) of each petrel colony during the breeding and non-breeding seasons are also depicted in the respective map. Black circles indicate the position of the colony. Background maps were created using the *maps* and *mapproj* packages of R (R Foundation for Statistical Computing, Vienna, Austria; www.r-project.org) and Adobe Illustrator (Adobe Systems Inc., CA, USA; www.adobe.com/products/illustrator.html).

**Figure S2.** Non-breeding probability maps for each of the Macaronesian gadfly petrels built using the respective breeding habitat models and the non-breeding environmental conditions (in a, b and c, for *P. madeira*, *P. deserta* and *P. feae*, respectively). Kernel density distributions (25, 50, 75 and 95%, from thicker to lighter line contours, respectively) of each petrel during the non-breeding seasons are also depicted in the respective map. Black circles indicate the position of the colony. Background maps were created using the *maps* and *mapproj* packages of R (R Foundation for Statistical Computing, Vienna, Austria; www.r-project.org) and Adobe Illustrator (Adobe Systems Inc., CA, USA; www.adobe.com/products/illustrator.html).

**REFERENCES**

1. Lebreton, J.-D., Burnham, K. P. ., Clobert, J. & Anderson, D. R. Modeling survival and testing biological hypotheses using marked animals: a unified approach with case studies. *Ecol. Monogr.* **62,** 67–118 (1992).

2. Choquet, R., Reboulet, A.-M., Pradel, R., Gimenez, O. & Lebreton, J.-D. *M-SURGE (Multi-state SURvival Generalized Estimation) 1.8 user’s manual*. (Centre d’Ecologie Fonctionnelle et Evolutive, CEFE-CNRS, 2006).

3. Choquet, R., Reboulet, A.-M., Lebreton, J.-D., Gimenez, O. & Pradel, R. U-CARE (Utilities-CApture-REcapture) 2.2 User’s Manual. (2005).

4. Burnham, K. P. & Anderson, D. R. *Model selection and model inference: a practical information-theoretic approach*. (Springer-Verlag, 1998).

5. Biotrack Ltd. United Kingdom. Available at: *www.biotrack.co.uk/m-series-bastrack-software.php*. Accessed 22 September 2015 (2015).

6. Phillips, R. A., Silk, J. R. D., Croxall, J. P., Afanasyev, V. & Briggs, D. R. Accuracy of geolocation estimates for flying seabirds. *Mar. Ecol. Prog. Ser.* **266,** 265–272 (2004).

7. R Development Core Team. *R: A language and environment for statistical computing*. (R Foundation for Statistical Computing). Available at: *www.rproject.org*. Accessed 14 April 2010 (2010).

8. Johnson, J. B. & Omland, K. S. Model selection in ecology and evolution. *Trends Ecol. Evol.* **19,** 101–108 (2004).

9. Zuur, A. F., Ieno, E. N., Walker, N. J., Saveliev, A. A. & Smith, G. M. *Mixed effects models and extensions in ecology with R*. (Springer, 2009).

10. Bates, D., Maechler, M. & Bolker, B. *Package lme4: Linear mixed-effects models using S4 classes. R package version 0.999375-42.* Available at: *cran.r-project.org* Accessed 13 December 2011 (2011).

11. Bartoń, K. *Package MuMIn: R functions for model selection and model averaging. R package version 1.6.5.* Available at: *cran.r-project.org*. Accessed 13 December 2011 (2011).

12. Shaffer, S. A., Costa, D. P. & Weimerskirch, H. Behavioural factors affecting foraging effort of breeding wandering albatrosses. *J. Anim. Ecol.* **70,** 864–874 (2001).

13. Wood, S. N. & Augustin, N. H. GAMs with integrated model selection using penalized regression splines and applications to environmental modelling. *Ecol. Modell.* **157,** 157–177 (2002).

14. Pinheiro, J. C. & Bates, D. M. *Mixed-Effects Models in S and S-PLUS*. (Springer Verlag, 2000).

15. Hastie, T. & Tibshirani, R. *Generalized Additive Models*. (Chapman & Hall/CRC, 1990).

16. Jacoby, W. G. Loess: a nonparametric, graphical tool for depicting relationships between variables. *Elect. Stud.* **19,** 577–613 (2000).

17. Phillips, S. J., Anderson, R. P. & Schapire, R. E. Maximum entropy modeling of species geographic distributions. *Ecol. Modell.* **190,** 231–259 (2006).
